# Fall Prevention Program Using Home Floor Plans in an Acute-Care Hospital: A Preliminary Randomized Controlled Trial

**DOI:** 10.3390/ijerph191711062

**Published:** 2022-09-04

**Authors:** Tetsuya Ueda, Yumi Higuchi, Tatsunori Murakami, Wataru Kozuki, Gentoku Hattori, Hiromi Nomura

**Affiliations:** 1 Course of Physical Therapy, Department of Rehabilitation Science, School of Medicine, Osaka Metropolitan University, Habikino City 583-8555, Osaka, Japan; 2 Department of Rehabilitation, Yao Tokushukai General Hospital, Yao City 581-0011, Osaka, Japan

**Keywords:** fall prevention, acute-care hospital, intervention study, discharged patients, home floor plans

## Abstract

We provided fall prevention programs using home floor plans for older adult patients discharged from an acute-care hospital and verified the fall prevention measures’ effectiveness six months after discharge. The research design was a preliminary randomized controlled trial. Orthopedic patients with a falls’ history were randomized to the control (*n* = 30) or the intervention groups (*n* = 30). Before discharge, the control group was treated with general physiotherapy for their disease characteristics. The intervention group received the same programs before discharge; additionally, a simple house evaluation was conducted using the subject’s home floor plan. A six-month follow-up survey was conducted on falls and near-falls after discharge, completed by 51 of the 60 subjects (85%). Within two months, falls occurred in 7.7% of the control group but not in the intervention group, after which, falls occurred in the intervention group, and no significant difference was noted between the two groups (three-month (*p* = 0.322) and six-month (*p* = 0.931) follow-ups). The intervention group had significantly fewer near-falls than the control group within three months (*p* = 0.034), but no significant difference was observed after three months. The results suggested that our program effectively expanded the role of an acute care hospital for discharged patients who need to transition from hospital care to home health care.

## 1. Introduction

Falls are typical among older people and hinder the preservation and improvement of the healthy life expectancy and quality of life of older adults. Patients discharged from acute-care hospitals [1,2,3,4] have a higher incidence of falls than community-dwelling older adults [5,6,7]. Furthermore, discharged patients encounter more frequent falls indoors than outdoors [3]. Falls often occur in places frequently used in daily life, such as the living room, bedroom, kitchen, and bathroom. Incidents such as stumbling or slipping (near-falls) are generally reported to be the causes of falls [8,9]. Near-falls are precursors to falls [10], but no reports have examined near-falls as precursors to falls in discharged patients.

Factors associated with falls are generally classified into two categories: internal and external factors. Interventions based solely on internal factors, those based solely on external factors, and, in recent years, those based on multiple factors from both categories have proven effective [11,12]. In the past, fall prevention programs in acute-care hospitals were based on internal factors such as lower limb muscle strengthening, dynamic balance, and applied walking exercises. Conversely, interventions based on external factors that seek to reduce fall risk, which is generally conducted by visiting and evaluating the patient’s house, were not considered feasible prevention methods. As a means of addressing external factors that contribute to falls, we evaluated the houses of patients discharged from acute-care hospitals based on their home floor plans [13]. The fall prevention program using home floor plans was effective for preventing falls during the first month after discharge. We consider that fall prevention programs using home floor plans and helping evaluate and treat a discharged patient’s physical function have high clinical utility for achieving a simple living environment that does not rely on home visits.

However, 40% to 50% of patients discharged to their homes fall within six months [4,14]. We need to clarify whether the effects of the fall prevention programs using home floor plans can be sustained for more than a month. To evaluate whether interventions based on external factors could reduce the risk of falls and near-falls, we examined the effectiveness of fall prevention programs before discharge using home floor plans for one month or longer after discharge.

## 2. Materials and Methods

### 2.1. Participants

Among older adults aged 65 years and older who were admitted to the orthopedic ward of an acute-care hospital, those who had a history of falls in the past year and were discharged home at the level of indoor independence (with or without walking aids) were included in this study. Patients with severe visual impairment who were scheduled for relocation or to be readmitted within six months were excluded from this study. Furthermore, to increase the reliability of home floor plans, those who scored below 24 on the cognitive function evaluation (Mini-Mental State Examination or MMSE) were also excluded. Subjects were recruited between 28 June 2014 and 15 August 2015 and followed until 15 August 2016.

### 2.2. Ethical Approval and Trial Registration

The purpose of this study was explained to all patients before acquiring their written consent. Moreover, this study was conducted with the approval of the Research Ethics Committee of the Graduate School of Comprehensive Rehabilitation, Osaka Prefecture University (approval number 2013-102). This study was registered in the University Hospital Medical Information Network Clinical Trials Registry (UMIN-CTR; UMIN000018201). We confirm that all related trials for this intervention are registered.

### 2.3. Research Design

The research design was a single-blind, preliminary randomized controlled trial. Consenting subjects were randomly assigned to the control and intervention groups through the permuted block method in batches of six. Each group included 30 subjects treated with the intervention until their discharge. In the assignment of the subjects, a third party who had no knowledge of the characteristics of the subjects used a random number generation program on a computer. The evaluators were trained by the research team, and the allocation was blinded.

We selected a sample size of *n* = 60 in this preliminary study. This value was chosen based not on formal statistical detection calculations but the value of choice in similar studies of fall prevention interventions [13,15]. The effect size of this study was 0.5 and statistical power was 0.8. We selected it to determine its potential for future large-scale studies.

### 2.4. Methods

Instructions were provided to each subject prior to and on the day of discharge. Prior to discharge, subjects in the control group were instructed to perform a voluntary training program as a form of general physiotherapy based on their disease characteristics. In particular, subjects who had undergone surgery for a proximal femoral fracture were instructed to perform muscle-strengthening exercises for the quadriceps femoris, iliopsoas muscle, and gluteus muscle groups. They were also instructed to perform one-leg standing balance training to the extent that the pain did not worsen. Voluntary training programs for fall prevention were provided to both groups. Additionally, in the intervention group, improvement methods for fall prevention according to the patient’s home were provided to address known fall risk factors based on the patient’s activity.

The tailored fall prevention programs, which included home floor plans, were provided to the participants by physical therapists who had less than two years of experience to remove the influence of the basic knowledge on fall prevention. Prior to giving out the programs, a trained physical therapist delivered a lecture on fall prevention knowledge and intervention methods. The lecture about fall prevention knowledge included the following points.

#### 2.4.1. Epidemiology of Falls among Older Adults

Factors contributing to falls among older adults are classified into internal and external factors [11,12]. Most falls occur indoors [3], and discharged patients [1,2,3,4] are at a high risk of falls.

#### 2.4.2. Evidence-Based Fall Prevention

The effective interventions for internal and external factors were explained using a handout. Additionally, it explained that initiatives addressing external factors conducted by physical and occupational therapists were effective in preventing falls and adjusting the living environment was also effective [16,17,18].

#### 2.4.3. Evaluation Method Based on the Home Floor Plan

The patients were told that their home floor plan would be used to check their activity revolving around the living room, where they spend most of their time during the day. The patients were also told a detailed interview would be conducted to confirm information not included in the home floor plan, such as steps, floor conditions, indoor wear, and illumination.

#### 2.4.4. Reducing Fall Risk Factors Confirmed from the Home Floor Plan

For known fall risk factors in the patients’ activities [11,16,17], they were advised regarding quick improvements, effective in preventing falls, based on their homes.

To unify qualitative and quantitative information regarding the intervention, we prepared a form that contained details of the instructions and where the participants could write the presence of fall risk factors. The instruction details emphasized the following: (1) illuminating steps, which are a fall risk factor, using fluorescent tape; (2) removing or fixing rugs or carpets to prevent slippage; (3) removing loose and slippery indoor footwear (e.g., slippers) or switching to fall-preventing slippers; (4) installing simple lighting fixtures, which do not require construction work, in dark places caused by lighting or structural problems; and (5) tidying places with objects such as papers or cushions scattered on the floor.

Lastly, a group of two people conducted a simulated patient instruction using a patient’s home floor plan.

### 2.5. Baseline Evaluation and Follow-Up

A baseline evaluation was conducted three days before discharge from the hospital, and an evaluation of cognitive function, activities of daily living (ADLs), physical function, mental and psychological function, and the preadmission life space were performed. Evaluations were performed using the MMSE for cognitive function, Barthel Index for the ADLs, the Timed Up and Go test (TUG) for physical function, Geriatric Depression Scale 5 (GDS5) and Modified Fall Efficacy Scale (MFES) for mental and psychological function, and Life-Space Assessment (LSA) for preadmission life space.

Additionally, the subject’s characteristics (age, sex, body mass index, illness, medical history, and medication status), history of falls, living environment, house environment, and walking ability prior to admission and at discharge were collected from their medical records.

A follow-up survey of indoor falls and near-falls was performed six months after discharge from the hospital. A fall was defined as “unintentionally falling to the ground or a lower level [19]”. On the other hand, a near-fall was defined as a slip, trip, or loss of balance (i.e., the individual starts to fall, but can stop or prevent the fall to the ground or a lower surface) [10,20,21]. Information about falls and near-falls was collected using a monthly falls calendar that subjects received through the mail. A phone interview was conducted for those who could not return the calendar.

### 2.6. Data Analysis

All analyses were conducted using the intention-to-treat principle. The baseline assessments of both groups were compared using a χ^2^ test and a *t*-test. Furthermore, a comparison of the incidence of falls and near-falls was conducted by including the time until the occurrence of falls or near-falls using the Kaplan–Meier method to calculate the percentage of those who did not experience falls or near-falls two months, three months, and six months after discharge. The statistical software program used was IBM SPSS ver 24.0 (IBM Japan, Tokyo, Japan), and the statistical significance level was 5%.

## 3. Results

### 3.1. Follow-Up of Subjects

**Figure 1:**
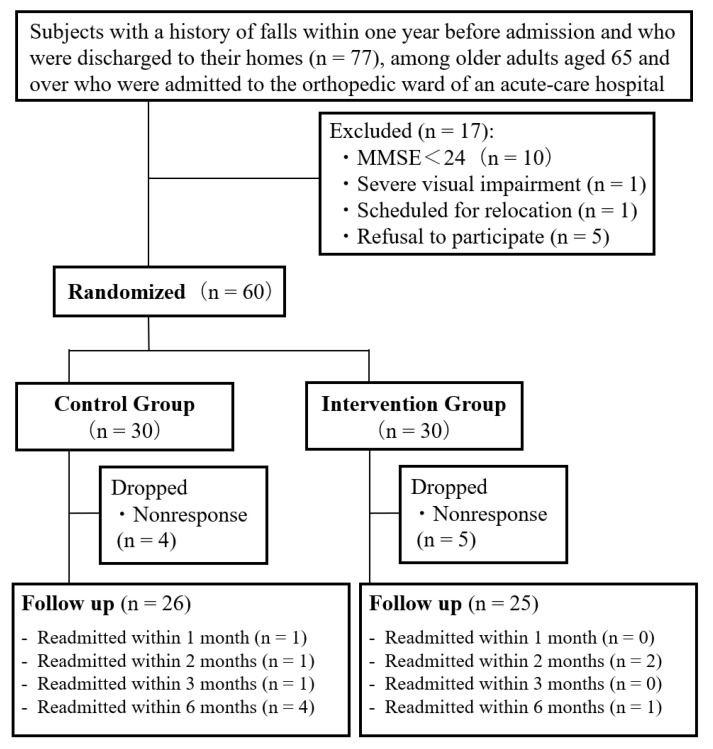
Assignment of subjects and flow chart. Note: MMSE, Mini-Mental State Examination.

The six-month follow-up survey was completed by 51 of the 60 subjects (85.0% follow-up rate; Figure 1). Four out of thirty subjects in the control group and five out of thirty subjects in the intervention group were excluded because they did not reply within one month of the follow-up, and we were unable to follow up and interview these subjects by phone. Additionally, we discontinued the follow-up of seven subjects in the control group and three subjects in the intervention group (ten subjects: 16.7%) due to readmission. The causes of readmission were as follows: one patient was readmitted in the first month (urinary tract infection), four patients by the third month (suppurative arthritis, laminoplasty, shunt construction, osteonecrosis), and five patients by the sixth month (joint deformity, mandibular osteomyelitis, necrosis of the femoral head, and locking plate removal in two patients). Readmissions of the three patients readmitted due to nonorthopedic diseases were unplanned.

### 3.2. Basic Characteristics of the Subjects and Survey Items

Table 1 presents the baseline comparison between both groups. The baseline evaluation before the intervention showed no statistically significant differences between the two groups in terms of primary characteristics and survey items. Additionally, no significant differences were observed between the two groups in terms of the lengths of hospitalization and rehabilitation and whether the reason for hospitalization was due to fall injury. As for comorbidities, 55 (91.7%) out of 60 had hypertension, diabetes, obstructive pulmonary disease, heart disease, stroke, tumor, or musculoskeletal disease.

### 3.3. Status of Fall Occurrence

**Figure 2:**
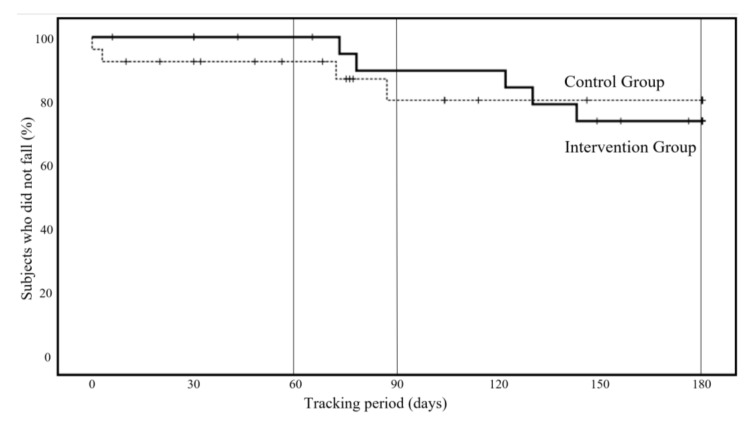
New occurrence of falls during the follow-up period (six months), Kaplan–Meier method.

Figure 2 shows the incidence of new falls during the follow-up period (6 months). There was no significant difference in the incidence of falls between the two groups.

In the control group, two patients (7.7%) had fallen by the second month of the follow-up period, whereas in the intervention group, no falls were recorded during the same period. However, from the second month onward, falls were also noted in the intervention group. As a result of the log-rank test using the Kaplan–Meier method, no significant differences were noted between the two groups in the analysis up to the third (*p* = 0.322, 95% confidence interval: 0.08 to 2.38) and sixth months (*p* = 0.931, 95% confidence interval: 0.25 to 3.55). No injuries such as fractures due to falls were noted.

At 60 days (two months) of follow-up, the incidence of falls was 7.7% in the control group and 0.0% in the intervention group. In the log-rank test, the *p*-value at 90 days (three months) was *p* = 0.322, and at 180 days (six months) it was *p* = 0.931.

### 3.4. Status of Near-Fall Occurrence

**Figure 3:**
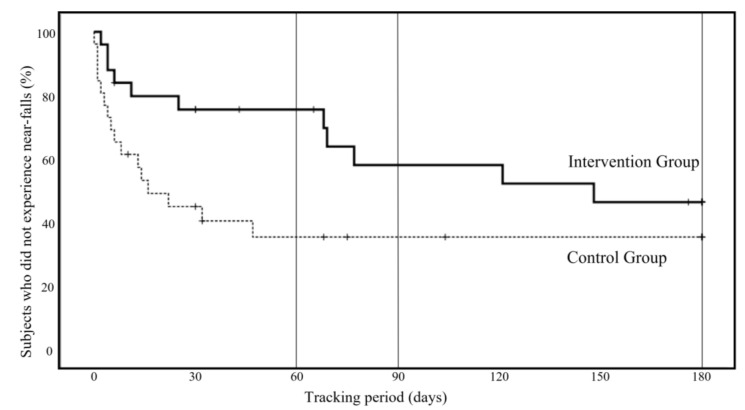
New occurrence of near-falls during the follow-up period (six months), Kaplan–Meier method.

Figure 3 shows the incidence of near-falls during the follow-up period (6 months). As a result of the log-rank test using the Kaplan–Meier method, the number of near-falls was significantly lower in the intervention group, based on the analysis up to the second month (*p* = 0.007, 95% confidence interval: 0.12 to 0.77). Furthermore, in the analysis up to the third month, the suppression of near-falls persisted in the intervention group (*p* = 0.034, 95% confidence interval: 0.19 to 0.97). However, a statistical suppression effect was not observed in the analysis up to the sixth month (*p* = 0.069, 95% confidence interval: 0.23 to 1.08).

## 4. Discussion

This study provided fall prevention instructions to patients [14,15] using home floor plans for older people admitted to the orthopedic ward of an acute-care hospital. Furthermore, we conducted a preliminary randomized controlled trial to verify the fall prevention effect within six months after discharge. As a result, although the fall prevention effect was observed for up to two months after discharge, a six-month effect was not observed.

The fall prevention effect was reported in this fall prevention program using home floor plans [13], but it was only described for one month after discharge. On the other hand, no effect on fall prevention was observed in individual programs aimed at providing fall prevention knowledge using digital video discs by a physical therapist for the hospitalized [14]. In this study, a follow-up survey was conducted six months after discharge. A fall prevention effect was observed for two months, and a near-fall prevention effect was described for three months. However, no effect was observed six months after discharge. The subjects of this study may have had yet to recover their physical functions after discharge sufficiently. It has been reported that it takes three months for ADLs to improve in a patient discharged from an acute-care hospital [22]. This study only recorded subjective reports of indoor falls, so no objective data are available to indicate whether the cause of the fall was mainly due to internal factors or external factors. However, we speculate that the subjects of this study had yet to sufficiently recover their physical functions three months after discharge. Therefore, we consider that an educational fall prevention intervention focused on the environmental factors that contribute to falls was effective in complementing the physical function of the subjects during the early stages of postdischarge recovery. Furthermore, the effect may have been suppressed since some subjects had fallen over the past year due to internal factors such as weakness in the lower limbs and psychological conditions. Nevertheless, no previous studies have reported a fall prevention effect that persisted beyond one month after discharge as a result of interventions based on internal factors, external factors, or multiple factors in instructions for fall prevention during discharge. Therefore, we consider that our study has sufficient clinical significance.

In this study, we conducted a comparative intervention trial to verify the fall prevention effect six months after discharge, and the six-month incidence of falls obtained was about 20% in the control group during the follow-up period. This result is lower than the 40–50% prevalence rate in previous studies [4,14]. This may be since previous studies targeted 80- to 85-year-old patients [4,14]. In contrast, the mean age in our study was around 75 years old, and the primary diseases of the subjects were orthopedic in nature. Additionally, the voluntary training instructions for orthopedic patients in the control group also included exercises such as muscle strengthening and balance training, effective for internal factors of fall risk. Therefore, we consider that the long-term incidence of falls may have been effectively suppressed.

On the other hand, in the intervention group, there was no occurrence of falls within two months after discharge. This result suggests that the efficacy of this study was confirmed early after discharge. Similarly, the effect for near-falls was observed within three months after discharge. Based on this finding, we consider that the intervention was effective in preventing near-falls early after discharge. The 2015 Nursing Care Benefit Revision Survey in Japan reported that 32% of people who used home services took two weeks or longer after discharge to start using home services. Meanwhile, 24% took one month or longer, and 16% took two months or longer [23] to start home services. The fall prevention effect was observed only up to two months after discharge from the results of this study. Home services must be started within two months after discharge to prevent refalling.

There are a few limitations to this study. First, the study was conducted in a single facility. This was because it has been elucidated that a single-center randomized controlled trial shows a higher therapeutic effect than its multicenter counterpart [24]. This study had few subjects, and it is considered a preliminary study. It is critical to advance intervention trials in multiple facilities moving forward; therefore, we are conducting multicenter studies. Second, we were unable to clarify whether the falls in this study were caused by environmental factors. However, since the subjects in this study had yet to recover their physical functions after discharge sufficiently, the internal factors may also have had a considerable impact on the occurrence of falls. Finally, the study was a single-blind study. However, we believe that a single-blind study was the most appropriate approach due to ethical considerations.

## 5. Conclusions

No effect was observed at six months for fall prevention using the patient’s home floor plan for discharge programs in acute-care hospitals. The tailored fall prevention programs before discharge that used home floor plans were influential in the early period after discharge. It is hoped that the contents of the intervention will be enhanced in the future to further extend the effects.

## Figures and Tables

**Table 1:** Baseline comparison between the control and intervention groups.

	Control Group (*n* = 30)	Intervention Group (*n* = 30)	*p* Value
Age (years)	76.7 ± 5.7	75.0 ± 7.0	0.291
Sex (no. of females, %)	21 (70.0)	20 (66.7)	0.781
Body mass index (kg/m^2^)	21.6 ± 3.9	22.9 ± 4.4	0.256
Primary disease (*n*, %)			0.563
-Upper limb disease	2 (6.7)	2 (6.7)	
-Lower limb disease	17 (56.7)	13 (43.3)	
-Trunk disease	11 (36.7)	15 (50.0)	
Comorbidity (*n*, %)			
Hypertension	10 (33.3)	17 (56.7)	0.069
Diabetes mellitus	6 (20.0)	7 (23.3)	0.754
Obstructive lung disease	3 (10.0)	1 (3.3)	0.306
Heart disease	6 (20.0)	4 (13.3)	0.365
Stroke	4 (13.3)	5 (16.7)	0.500
Tumor	4 (13.3)	5 (16.7)	0.500
Motor disorders (aside from primary disease)	14 (46.7)	17 (56.7)	0.438
Medication status (*n*, %)	6 (20.0)	4 (13.3)	0.365
MMSE (points)	27.3 ± 2.0	27.5 ± 2.2	0.715
Fall injury causing hospitalization	22 (73.3)	18 (60.0)	0.273
History of frequent falls (*n*, %)	7 (23.3)	5 (16.7)	0.519
Living environment; cohabitant (*n*, %)	20 (66.7)	20 (66.7)	1.000
House environment; detached house (*n*, %)	25 (83.3)	24 (80.0)	0.739
LSA (points)	78.2 ± 24.4	77.0 ± 23.4	0.851
Walking ability prior to admission; independent walking (*n*, %)	27 (90.0)	27 (90.0)	0.665
Walking ability at discharge; independent walking (*n*, %)	12 (40.0)	15 (50.0)	0.436
Barthel Index (points)	96.8 ± 5.5	97.7 ± 4.7	0.530
TUG (seconds)	19.2 ± 13.9	17.0 ± 10.6	0.508
GDS5 (points)	1.9 ± 1.2	1.9 ± 1.5	0.926
MFES (points)	108.3 ± 22.4	107.9 ± 29.6	0.949
Length of hospital stay (days)	23.5 ± 13.7	25.0 ± 16.3	0.694
Length of rehabilitation (days)	19.1 ± 13.4	20.2 ± 15.2	0.761

Notes. The mean value ± standard deviation or the number of people (%). Upper limb diseases include humeral fractures and scapula fractures. Lower limb diseases include femoral fractures, tibial fractures, patellar fractures, and artificial knee joints. Trunk diseases include spinal compression fractures, spinal canal stenosis, and spinal cord injury. The medication status shows if patients were taking psychotropic drugs, benzodiazepine drugs, or antidepressants. MMSE, Mini-Mental State Examination (the score range is 0–30); LSA, Life-Space Assessment (the score range is 0–120); TUG, Timed Up and Go test; GDS5, Geriatric Depression Scale 5 (the score range is 0–5); MFES, Modified Fall Efficacy scale (the score range is 0–140).

## Data Availability

The data presented in this study are available on request from the corresponding author. The data are not publicly available due to restrictions regarding privacy or ethics.
